# The study of the association of the p35 rs17852832 SNP polymorphism with Alzheimer’s disease

**DOI:** 10.1186/1750-1326-7-S1-S9

**Published:** 2012-02-07

**Authors:** Lili Shi, Yihua Qian, Xiaodan Hu, Hua Han, Yong Liu

**Affiliations:** 1 Department of Human Anatomy and Histology Embryology, Institute of Neurobiology, Key Laboratory of Environment and Genes Related to Diseases of Education Ministry, Xi’an Jiaotong University College of Medicine, China

## Background

Alzheimer’s disease (AD) may be caused by multiple factors, including genetics, age, environment etc. At present, AD associated genes are: gene *App*, *ps1*, *ps2* and *apoE*. However, these associated genes account mainly for the abnormal increase and accumulation of Aβ, rather than the molecular genetic mechanism for the formation of neurofibrillary tangles and neuronal loss. P35 is a neuron specific regulative unit of CDK5. *p35* gene contain several SNPs, and at some SNPs sites, the change of a single base results in the corresponding change of P35 amino acids. Cleavage of P35 into P25 greatly increases the kinase activity of CDK5, which in turn abnormally phosphorylates tauprotein, and then contributes to the formation of neurofibrillary tangles. What we are interested in is whether the polymorphism of the P35 gene was involved in the pathogenesis of AD. There are few research reports about the relationship of the P35 gene polymorphism with AD.

## Methods

To explore the association of the *p35* rs17852832 polymorphism with AD, molecular biologic techniques were adopted, such as polymerase chain reaction (PCR), the analytical method of restriction fragment length polymorphism (RFLP) etc, and the frequency of genotypes and alleles of the *p35* rs17852832 polymorphism in AD and normal senile people were analyzed.

## Results

1. The PCR products were examined through 2% agarose gel electrophoresis, and found that closely after the 250bp band existed a regular and clear band, which was the intended band of 260bp fragments of *p35* gene including rs17852832 SNP. 

2. The purified fragments of 681-940bp of *p35* gene including rs17852832 SNP were analyzed with method of RFLP, and found that the *p35* gene fragments of all cases of AD and controls were cut by Mva I into three shorter fragments, which length were 114bp, 90bp and 53bp. The allele and the genotype of SNP rs17852832 were C and C/C respectively, allele A and genotype C/A or A/A hadn’t been found yet. 

3. The purified and identified fragments of 681-940bp of *p35* gene were cloned into pMD18-T vector for sequencing. The sequencing result showed that they were identical to the *p35* gene sequence from GENBANK, which show that the sequence cloned into T vector was our intended fragments, *p35* gene 681-940bp including rs17852832 SNP. And that, the sequencing result proved further that the allele and genotype of rs17852832 SNP were C and C/C respectively, allele A and genotype C/A or A/A hadn’t been found yet.

## Conclusion

The RFLP of *p35* gene 681-940bp fragments were analyzed through restrictive enzymes and PAGE technique, and made sure that the allele of SNP at 798 site of *p35* gene in all cases of AD and controls were C, which was identical to sequence provided by GENBANK; Genotype of rs17852832 SNP were C/C, allele A and genotype C/A or A/A hadn’t been found yet.

